# A clinical prediction model for blood pressure changes after renal denervation in patients with resistant hypertension

**DOI:** 10.3389/fcvm.2025.1637388

**Published:** 2025-07-21

**Authors:** Yishuan Zhang, Ruiqing He, Chen Chen, Hong Zhang, Lingyan Li, Rongxue Xiao, Shuangyu Chen, Shuyi Wu, Zongjun Liu, Junqing Gao

**Affiliations:** ^1^ Shanghai Putuo Central School of Clinical Medicine, Anhui Medical University, Shanghai, China; ^2^ Department of Cardiology, Putuo Hospital, Shanghai University of Traditional Chinese Medicine, Shanghai, China

**Keywords:** renal denervation, clinical prediction model, resistant hypertension, index of microvascular resistance, blood pressure change

## Abstract

**Objective:**

To develop clinical prediction models to estimate blood pressure changes in hypertensive patients undergoing renal denervation (RDN).

**Methods:**

This single-center, prospective interventional study enrolled 70 hypertensive patients undergoing RDN between July 2022 and December 2023, with clinical data collected systematically before and after the procedure. Variable selection for modeling was performed through a rigorous process incorporating univariate analysis and clinical relevance assessment. Subsequently, two distinct clinical prediction models were developed and subjected to comparative evaluation. The primary outcomes were the absolute changes in systolic blood pressure (SBP) and diastolic blood pressure (DBP) at 6 months after RDN.

**Results:**

In both Ordinary Least Squares (OLS) and Ridge regression models, seven variables [including index of microvascular resistance (IMR), preoperative SBP, age and creatinine] were significantly associated with SBP change, while four variables were significantly associated with DBP change. In the prediction model on SBP change, compared to the OLS model, the Ridge regression exhibited lower prediction errors [mean absolute error [MAE]: 6.40 vs. 6.95; mean squared error [MSE]: 65.58 vs. 76.15] and a higher R² (0.79 vs. 0.72). In the DBP model, the Ridge regression also achieved a lower MAE (3.62 vs. 3.73) and a higher R² (0.77 vs. 0.71).

**Conclusion:**

This study developed and compared predictive models for estimating blood pressure response at 6-month follow-up after RDN in patients with resistant hypertension. The Ridge regression model exhibited superior predictive accuracy and model stability. The model indicated that IMR was a factor associated with postoperative blood pressure reduction.

**Clinical Trial Registration:**

ClinicalTrials.gov, identifier, ChiCTR2200058696.

## Introduction

Hypertension has long been a chronic health challenge causing cardiovascular events globally, affecting over 1.5 billion people worldwide. Currently, the basic methods treating hypertension involves a combination of medication and lifestyle improvements. In recent years, Renal Denervation (RDN) has gained significant attention as a novel method on hypertension treatment. Despite ongoing debates about its efficacy and safety since its introduction (1) with the deepening of research, a series of clinical evidence has accumulated, strongly supporting the long-term, stable blood pressure reduction effectiveness and safety of RDN (2–4). With technological advances and in-depth clinical practices, ultrasound RDN (uRDN, Recor Paradise) was approved by the U.S. Food and Drug Administration (FDA) on November 7, 2023. Several weeks later, multi- electrode radiofrequency RDN (Medtronic Spyral) also received FDA approval and was recently launched in China. The principle of RDN lies in selectively removing renal sympathetic nerves so as to lower blood pressure. It is particularly suitable for patients whose blood pressure remains poorly controlled despite lifestyle changes and medication (5–7). Although the effectiveness of RDN in reducing blood pressure has been recognized and is gradually being used globally, it still faces several challenges in practice. Currently, due to the lack of a clear and unified standard for determining surgical endpoints, surgeons often find it difficult to accurately judge when the optimal therapeutic effect is achieved during surgery, which may lead to excessively long or short surgical time, too many or too few ablation sites, thereby affecting patient recovery and treatment effectiveness (8). Our research team observed a significant dilation of the renal artery diameter post-surgery in previous studies. We also attempted to apply Fractional Flow Reserve (FFR) in RDN procedures and founded that renal artery FFR could assess the immediate effectiveness of RDN (9). However, due to additional operation time, cost, and technical complexity, it is difficult to implement FFR on a large scale. The coronary angiography-derived index of microcirculatory resistance (caIMR), based on three-dimensional quantitative coronary angiography using computational fluid dynamics analysis, calculates coronary microcirculatory resistance using FlashAngio software (10). FlashAngio is currently mainly used for coronary function assessment, and there is preliminary data on its application in pan-vascular areas, especially in renal and pulmonary vessels. Based on the calculation principles of caIMR, we have derived the renal artery index of microcirculatory resistance (raIMR). This study aims to evaluate renal microcirculatory resistance using this indicator and to explore the quantitative relationship between the renal microcirculatory resistance index and the efficacy of percutaneous RDN in the treatment of hypertension. Furthermore, combined with other indicators affecting blood pressure, a clinical prediction model was constructed to predict the change of blood pressure in hypertensive patients after RDN.

## Subjects and methods

### Subjects

This is a single-center prospective interventional study (clinical trial registration number: ChiCTR2200058696), which included 71 hypertensive patients who underwent RDN treatment at Putuo District Central Hospital in Shanghai from July 2022 through December 2023.

Inclusion criteria:

1. Age: over 18 years old
2. Disease status: Resistant hypertension
3. Treatment status: Post-RDN surgery
4. Consent to participate: Voluntary participation with signed informed consent, willing to follow up later.

Exclusion Criteria:

1. Renal artery angiography shows ≥50% stenosis on either side;
2. Systolic blood pressure ≤90 mmHg;
3. The indication for RDN surgery is not hypertension;
4. Participation in other clinical studies.

### Interventions

RDN treatment process: Take 300 mg of aspirin and 300 mg of clopidogrel 24 hours before the procedure. During the surgery period, administer 50 U/kg of heparin intravenously. After puncturing the femoral artery and placing a 7F vascular sheath, perform renal artery angiography. After confirming no stenosis or other lesions in the left and right renal arteries, place an RDC catheter at the ostium of the left and right renal arteries. Then, irrigate a 5F radiofrequency ablation catheter (HDA5C090TC, Shanghai Huida Medical Device Co., Ltd.) with physiological saline and perform radiofrequency ablation on the renal artery. Perform circumferential ablation on the trunks of the left and right renal arteries, with each ablation site lasting for 60 s and spaced 5 mm apart. Recheck the renal artery angiography postoperatively.

### Data collection

During the study period, the following data were recorded for each patient: gender, age, smoking history, medical history (coronary heart disease, myocardial infarction, cerebral infarction), preoperative and postoperative office SBP and DBP, and preoperative and postoperative renal artery index of microcirculatory resistance (raIMR) for the left and right sides [preoperative left side recorded as raIMR(Pre-LRA), preoperative right side recorded as raIMR(Pre-RRA), postoperative left side recorded as raIMR(Post-LRA), postoperative right side recorded as raIMR(Post-RRA)], as well as the changes in bilateral renal artery IMR [raIMR(Post-LRA)-raIMR (Pre-LRA)] + [raIMR(Post-RRA)-raIMR(Pre-RRA)]. Additionally, changes in renal artery diameter before and after RDN surgery (postoperative renal artery diameter—preoperative renal artery diameter) [including the first renal artery (left kidney), first renal artery (right kidney), second renal artery (left kidney), second renal artery (right kidney)] were collected. Preoperative laboratory parameters included white blood cell count (10^9/L), platelet count (10^9/L), hemoglobin (g/L), alanine aminotransferase (ALT, U/L), aspartate aminotransferase (AST, U/L), creatinine (μmol/L), uric acid (μmol/L), blood urea nitrogen (BUN, mmol/L), blood glucose (mmol/L), glycated hemoglobin (HbA1c, %), total cholesterol (mmol/L), low-density lipoprotein (LDL, mmol/L), high-density lipoprotein (HDL, mmol/L), and preoperative left ventricular ejection fraction (LVEF, %).

### raIMR measurement

To calculate the renal artery index of microcirculatory resistance (raIMR), we used a formula similar to the coronary artery index of microcirculatory resistance (caIMR). The formula for raIMR is given by:

$$\mathrm{raIMR}=P_{d}\cdot \frac{\;L\;}{\;K\cdot V_{\mathrm{average}\;}\;}$$

where:

• V_average_ is the mean blood flow velocity in the renal artery (mm/s),
• L is the length of the renal artery from the ostium to the distal end (mm),
• P_d_ is the mean pressure at the distal end of the renal artery (mmHg),
• K is a constant.

The final results were calculated using the FlashAngio software and it was used to represent the IMR of the renal artery in this study. The calculation principle of the raIMR for the renal artery is similar to that of the coronary artery, but constant K (related to vascular elasticity, viscosity coefficient, and density), vessel length L, and distal pressure P_d_ are all closely related to the physiological functions of the renal vessels themselves.

### Outcome measures

In this study, the primary outcome measures were the changes in systolic blood pressure (SBP change) and diastolic blood pressure (DBP change) at the 6-month follow-up. These were calculated as the difference in the blood pressure between the 6-month follow-up and the preoperative baseline.

## Results

### Baseline characteristics

**Figure 1:**
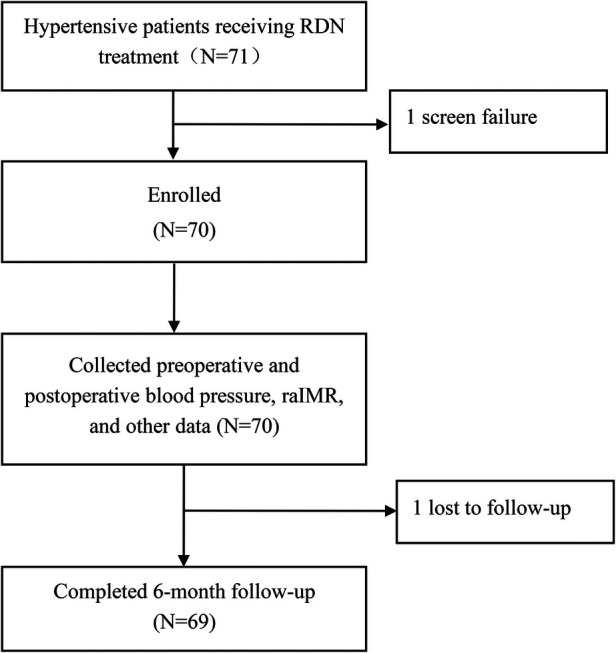
The picture is a flow chart of the study. The total number of patients initially enrolled was 71, of which 2 patients failed to complete the follow-up, and the final number of study was 69.

In this study, a total of 71 patients with resistant hypertension who underwent RDN treatment were screened. Among them, one patient did not meet the inclusion criteria and another patient did not complete the follow-up. Ultimately, 69 patients completed the follow-up and their data were analyzed (Figure 1). Their baseline characteristics were summarized in Table 1. The mean ± standard deviation (SD) age was 65.6 ± 11.83 years, and 54 (78.3%) were male. The mean preoperative office SBP and DBP values were 153.2 ± 19.85 mmHg and 84.6 ± 12.01 mmHg, respectively.

**Table 1:** Baseline characteristics.

Characteristics	Overall patients (*n* = 69)
Age (years)	65.6 ± 11.83
Gender, male	54 (78.3%)
History of coronary heart disease	31 (44.9%)
History of myocardial infarction	24 (34.8%)
History of cerebral infarction	6 (8.7%)
History of smoking	18 (26.1%)
SBP (mmHg)	153.2 ± 19.85
DBP (mmHg)	84.6 ± 12.01
Laboratory findings	
WBC (10^9/L)	7.60 ± 2.41
PLT (10^9/L)	204.3 ± 58.19
HB (g/L)	136.6 ± 19.16
ALT (U/L)	26.5 ± 17.24
AST (U/L)	23.9 ± 9.07
Cr (μmol/L)	91.3 ± 36.37
UA (μmol/L)	413.0 ± 113.47
BUN (mmol/L)	9.6 ± 12.20
Glucose (mmol/L)	6.2 ± 2.15
HbA1c (%)	6.4 ± 1.05
TC (mmol/L)	4.4 ± 1.28
TG (mmol/L)	1.8 ± 2.23
LDL (mmol/L)	2.9 ± 0.96
HDL (mmol/L)	1.0 ± 0.25
LVEF (%)	51.4 ± 14.02

Data are presented as mean ± SD or *n* (%). ALT, alanine transaminase; AST, aspartate transferase; BUN, blood urea nitrogen; Cr, creatinine; DBP, diastolic blood pressure; HB, haemoglobin; HbA1c, glycated haemoglobin; HDL, high-density Lipoprotein; LDL, low-density Lipoprotein; LVEF, left ventricular ejection fraction; PLT, platelets; SBP, systolic blood pressure; SD, standard deviations; TC, total cholesterol; TG, triglyceride; UA, uric acid; WBC, white blood cell.

### Development of prediction models

#### Selection of potential predictors

Before constructing the clinical prediction models, potential predictive factors that might influence outcome variables (SBP change and DBP change) were strictly screened with the use of statistical analyses. For continuous variables, simple linear regression analysis was used to calculate *p*-values and coefficient of determination (R^2^). For categorical variables, independent sample *t*-test was performed to calculate *p*-values. Preliminary results showed that cerebral infarction, IMR, preoperative SBP (PreSBP), preoperative DBP (PreDBP), aspartate transferase, creatinine, total cholesterol, and low-density lipoprotein (LDL) were significantly associated with SBP change (all *P* < 0.05). The variables significantly associated with DBP change included age, IMR, PreDBP, creatinine, total cholesterol, and LDL (all *P* < 0.05). The definitions and results of each predictive variable are detailed in Supplementary Tables 1, 2.

To avoid including variables with weak predictive power, we further evaluated the explanatory power of each variable based on R². Variable selection was also guided by clinical significance and practical relevance. Finally, the SBP change prediction model included cerebral infarction, IMR, PreSBP, age, creatinine, total cholesterol and LDL as predictive factors, while the DBP change prediction model included IMR, PreDBP, age and creatinine level.

#### Model development

In this study, SBP change and DBP change were used as the primary outcome indicators to separately construct prediction models. To explore the performance of different statistical modeling methods, we employed two regression models: Ordinary Least Squares (OLS) regression and Ridge regression.

#### Relationship between predictors and outcomes

**Figure 2:**
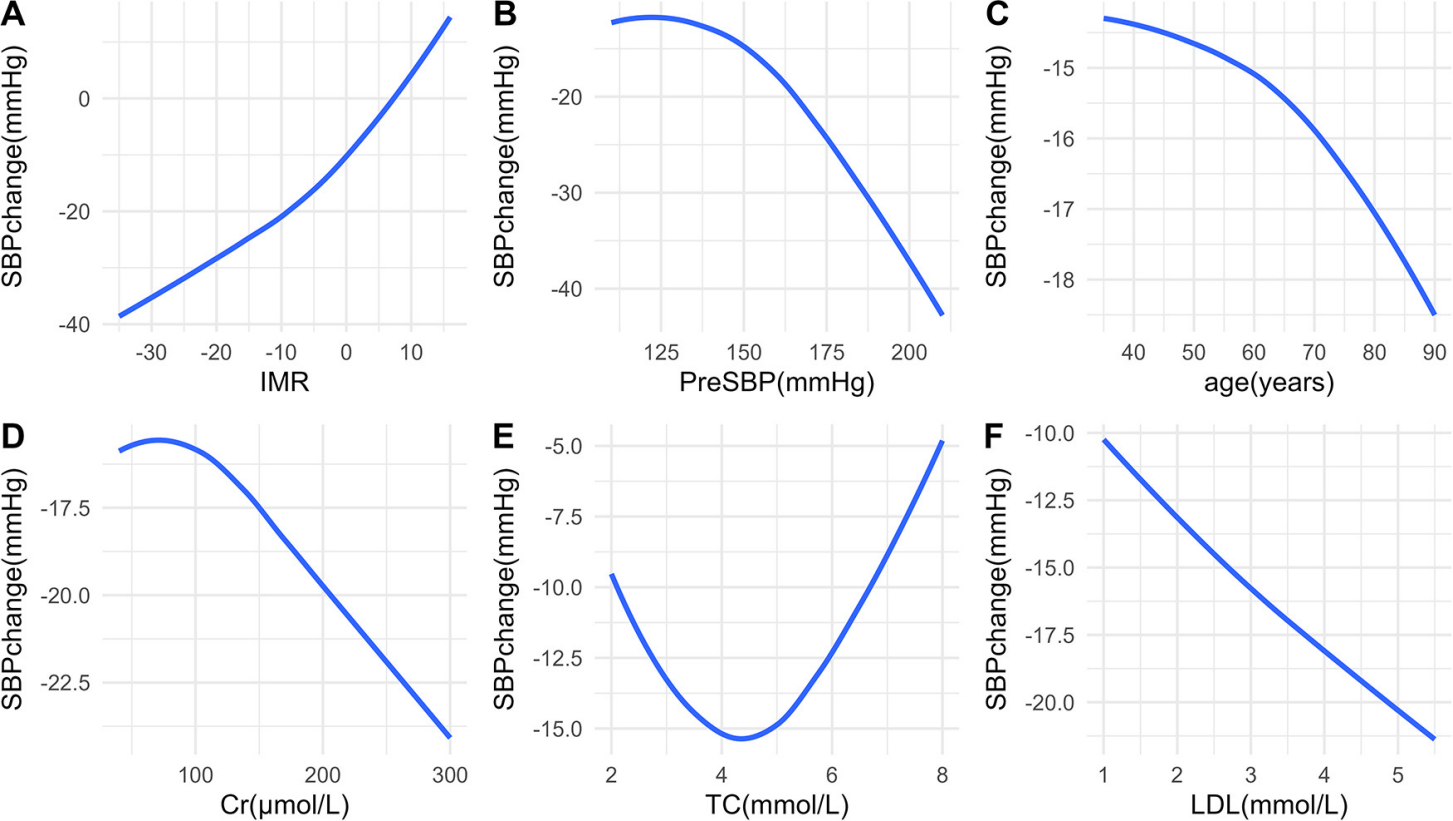
Relationships between predictors and SBP change. Associations between SBP change and IMR **(A)**, PreSBP **(B)**, age **(C)**, Cr **(D)**, TC **(E)**, and LDL **(F)** SBP, systolic blood pressure; IMR, index of microcirculatory resistance; PreSBP, preoperative systolic blood pressure; Cr, creatinine; TC, total cholesterol; LDL, low-density lipoprotein.

**Figure 3:**
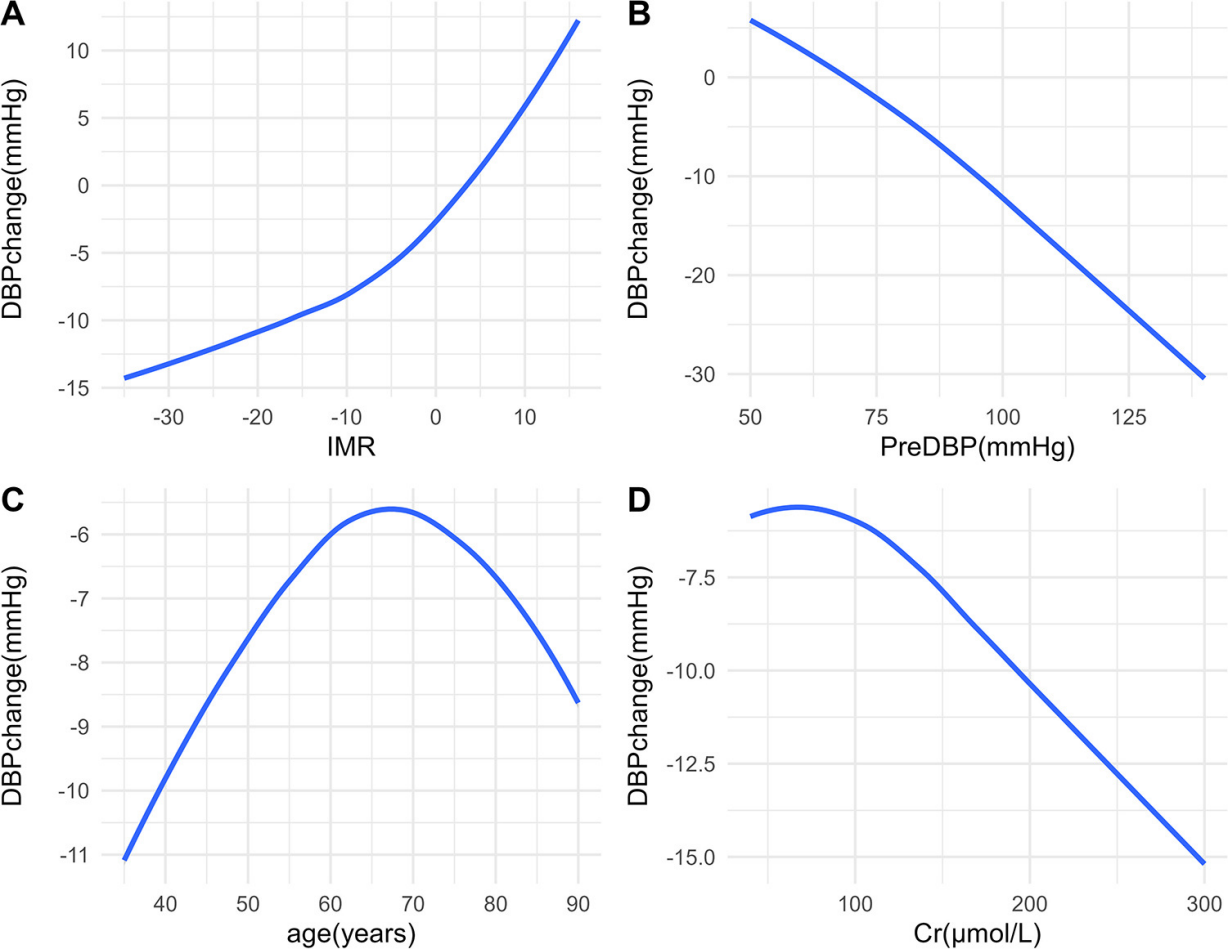
Relationships between predictors and DBP change. Associations between DBP change and IMR **(A)**, PreDBP **(B)**, age **(C)**, and Cr **(D)** DBP, diastolic blood pressure; IMR, index of microcirculatory resistance; PreDBP, preoperative diastolic blood pressure; Cr, creatinine.

In the OLS model, we used spline regression to plot the relationship curves between each predictive variable and the outcome variables (SBP change and DBP change) (Figures 2, 3). The results showed that a greater decrease in IMR was associated with a larger reduction in both SBP (Figure 2A) and DBP (Figure 3A). Patients with higher preoperative SBP and DBP experienced greater blood pressure reductions after RDN surgery (Figures 2B, 3B). As age increased, there was a trend toward a greater reduction in SBP (Figure 2C), which may be due to higher baseline SBP in older patients, providing greater potential for blood pressure reduction. However, DBP change exhibited a nonlinear trend (Figure 3C). Additionally, creatinine levels were inversely associated with changes in both SBP and DBP, with higher creatinine levels corresponding to greater reductions in blood pressure (Figures 2D, 3D). A U-shaped relationship was observed between TC and SBP change, indicating that both high and low cholesterol levels may influence blood pressure reduction (Figure 2E). LDL levels were negatively correlated with SBP change, with higher LDL levels being associated with greater reductions in SBP (Figure 2F).

#### Model performance

**Figure 4:**
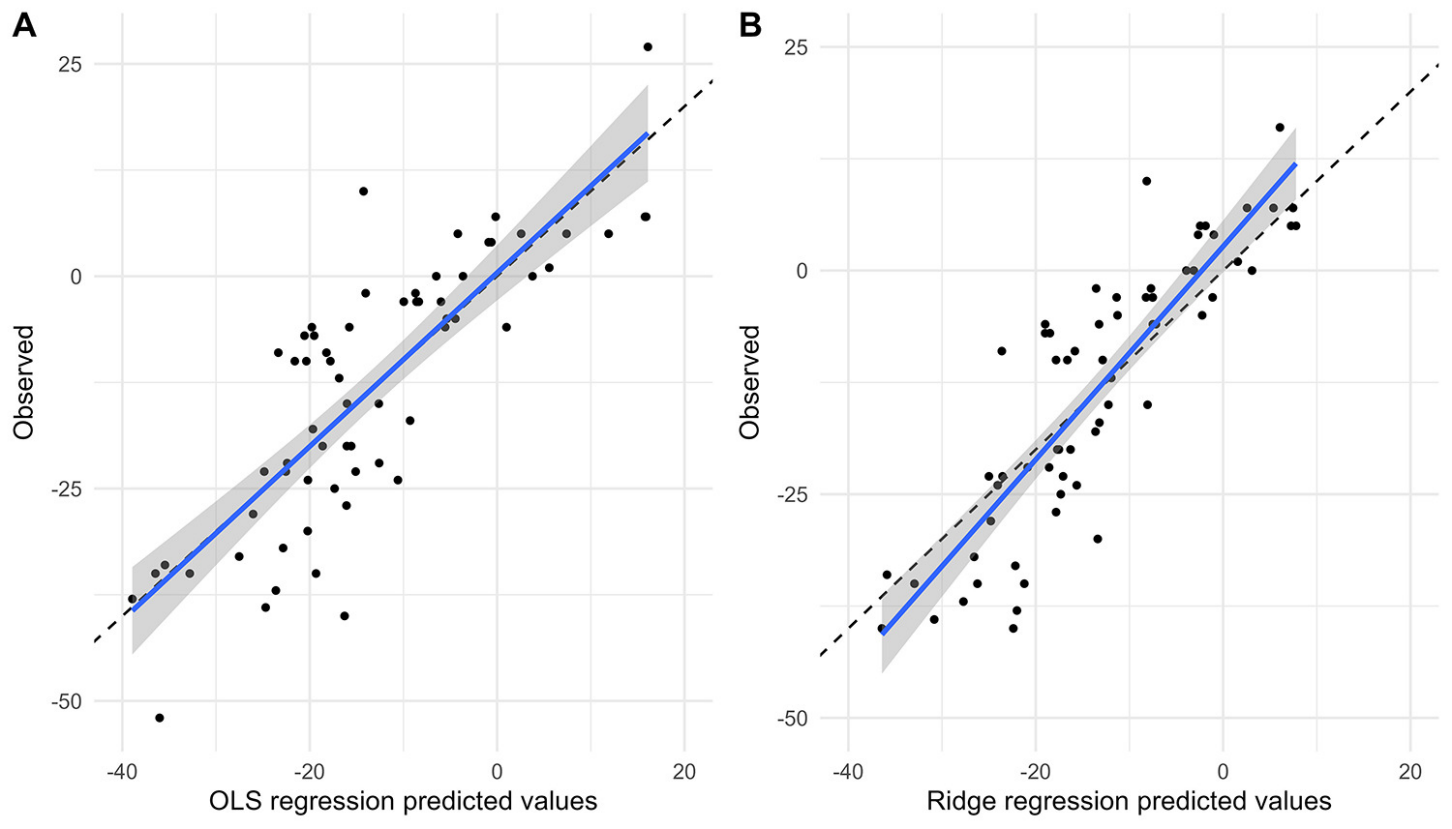
Comparison of predicted and observed values of SBP change. Comparison of predicted and observed values from OLS model **(A)** and Ridge regression model **(B)** for SBP change at 6 months.

**Figure 5:**
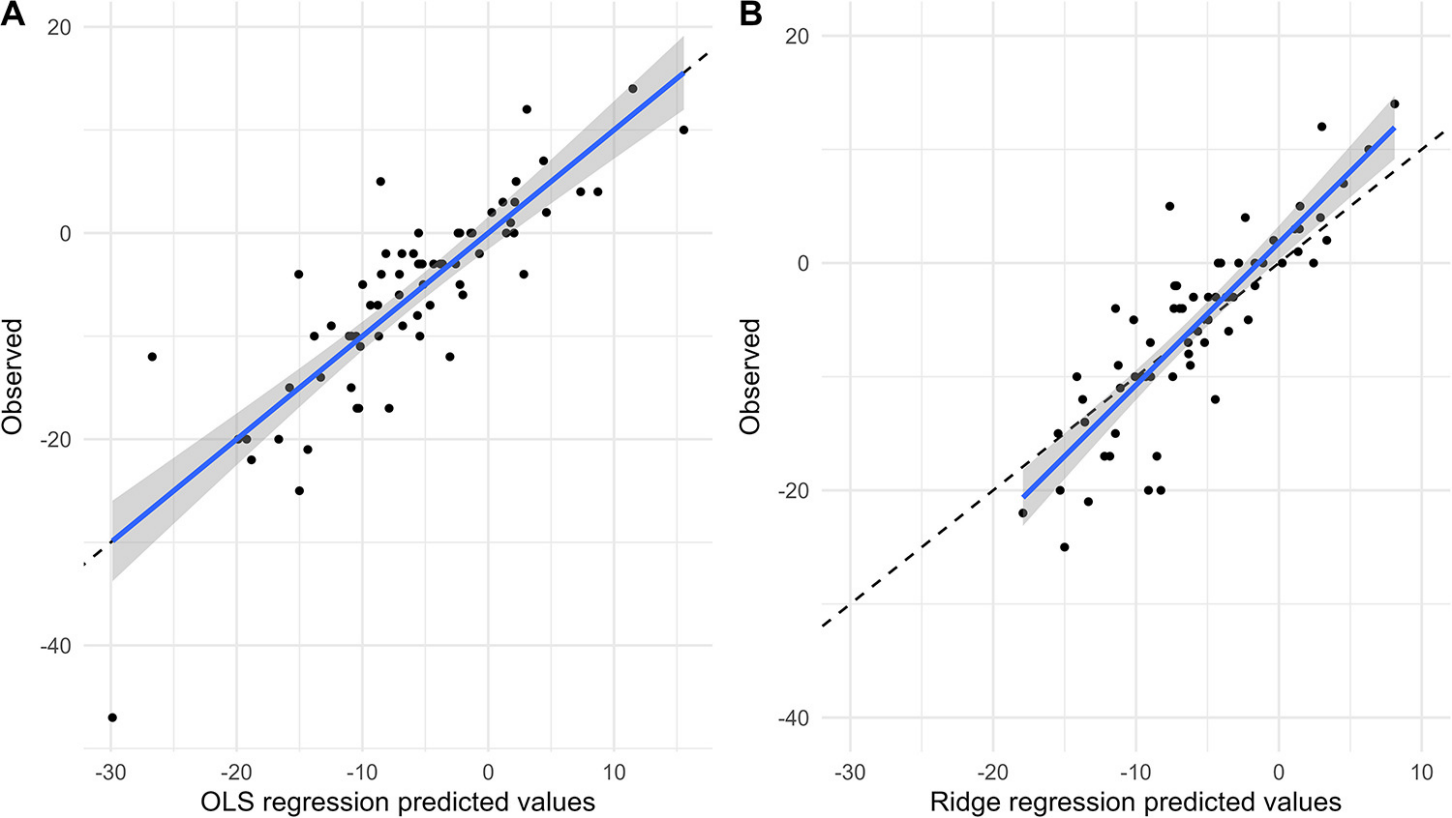
Comparison of predicted and observed values of DBP change. Comparison of predicted and observed values from OLS model **(A)** and Ridge regression model **(B)** for DBP change at 6 months.

Figures 4, 5 show the comparison between predicted and observed values for SBP change and DBP change in the OLS regression model and the Ridge regression model, respectively. As shown in these two figures, the OLS model exhibits a significant discrepancy between predicted and observed values, particularly in the lower prediction range, where it shows larger bias and poorer accuracy. In contrast, the Ridge regression model demonstrates smaller bias across the entire prediction range, with predicted values more closely clustered around the observed values. This is particularly noticeable in cases of extremely high or low predicted values, where its predicted values aligned more closely with the actual observed ones.

#### Model comparison

**Figure 6:**
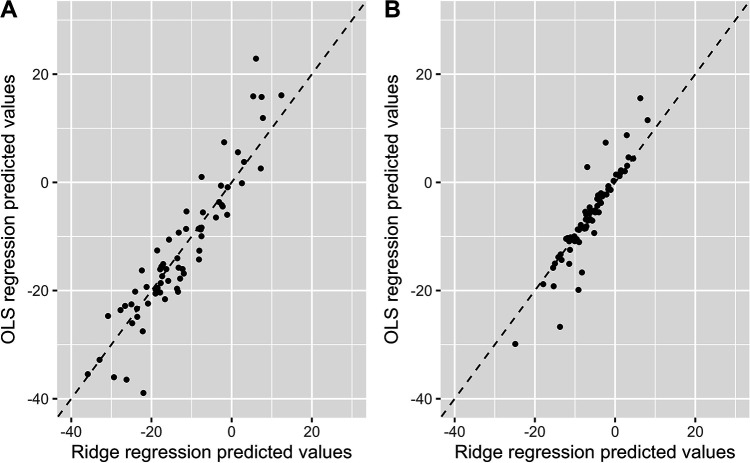
Comparison of the predicted values between the OLS model and the ridge regression model. Comparison of OLS and Ridge regression models predicting SBP change **(A)** and DBP change **(B)** OLS, ordinary least squares; SBP, systolic blood pressure; DBP, diastolic blood pressure.

Figure 6 illustrates the relationship between the predicted values from the OLS model and from the Ridge regression model. Overall, most points are concentrated near the diagonal line, indicating that the predictions from both models are largely consistent. However, at higher or lower predicted values, inconsistencies between the two models are observed. In these cases, the Ridge regression model demonstrates greater robustness with less variability in its predictive values.

#### Model performance evaluation

We evaluated the performance of the OLS model and the Ridge regression model in predicting blood pressure change after RDN using mean absolute error (MAE), mean squared error (MSE), and coefficient of determination (R²). In the prediction model on SBP change, compared to the OLS model, the Ridge regression model exhibited lower prediction errors (MAE: 6.40 vs. 6.95; MSE: 65.58 vs. 76.15) and a higher R² (0.79 vs. 0.72), indicating a better fit and improved explanatory power. In the DBP model, although the Ridge regression model had a slightly higher MSE (26.92 vs. 26.23), it achieved a lower MAE (3.62 vs. 3.73) and a higher R² (0.77 vs. 0.71), suggesting that Ridge regression offers an advantage in reducing prediction error and enhancing model interpretability.

Overall, the Ridge regression model showed superior performance in predicting blood pressure change after RDN.

## Discussion

RDN originated in the early 1930s when surgeons Smithwick and Thompson discovered that the sympathetic nervous system was generally overactive in most patients with high blood pressure. They attempted to treat hypertension by surgically removing the sympathetic trunk on the thoracolumbar spine and the splanchnic nerve emanating from the celiac ganglion. However, this approach was abandoned due to complications and the rise of antihypertensive drugs (11, 12). It wasn't until 2009 that Murray Esler's team completed the Simplicity HTN-1 and Simplicity HTN-2 clinical trials, which showed a reduction in office systolic blood pressure (SBP) of 22 mmHg and 32 mmHg, respectively, at the six-month follow-up (13, 14). However, the Simplicity HTN-3 clinical trial, led by Medtronic in 2012, did not yield the expected results (15). In recent years, RDN has regained momentum. For instance, the RADIANCE II study, which is based on ultrasound catheters, showed that renal denervation with ultrasound could reduce daytime ambulatory SBP at two-month post-op follow-up visit, even without antihypertension medications (16). In a long-term efficacy and safety observational trial (SPYRAL HTN-ON MED) that performed renal artery ablation alongside antihypertensive drug therapy, no short-term or long-term safety issues related to renal denervation were found after 36 months of follow-up (17). However, RDN still faces a series of challenges, such as unclear patient selection criteria and insufficient detection of intervention endpoints. New technologies have emerged in response, such as the electrical stimulation of renal artery autonomic nerves, which can increase blood pressure by increasing central sympathetic nerve activity. This response can be used to determine the target ablation site and endpoint for RDN (18); three-dimensional reconstruction technology combined with renal nerve stimulation-guided radiofrequency catheter ablation can also promote the application of selective and precise RDN in actual clinical practice (19); the use of saline-flush radiofrequency ablation (RFA) catheters, which are commonly used for cardiac tissue ablation, can also improve the safety and effectiveness of RDN (20).

This study developed a prediction model for the reduction in blood pressure 6 months after RDN in hypertensive patients and identified several variables significantly associated with the magnitude of blood pressure reduction, including cerebral infarction, IMR, preoperative SBP, age, creatinine, total cholesterol, and low-density lipoprotein. Among these variables, the reduction in IMR was positively correlated with the reduction in blood pressure, meaning that the greater the postoperative reduction in IMR, the more significant the blood pressure reduction. IMR reflects changes in renal artery microcirculation resistance, suggesting that RDN may exert its antihypertensive effect by improving microcirculation function. Furthermore, creatinine levels were negatively correlated with both SBP and DBP change, indicating that patients with impaired kidney function may benefit more from RDN, especially when creatinine levels exceed the normal range, leading to more significant blood pressure reduction. This could be related to higher sympathetic nervous system activity and excessive activation of the renin-angiotensin-aldosterone system (RAAS) in patients with kidney dysfunction, making the antihypertensive effect of RDN more pronounced.

Additionally, this study found that TC and LDL levels may also influence the extent of blood pressure reduction, which is consistent with previous studies that indicated a relationship between LDL and total cholesterol levels and post-RDN blood pressure changes (21–23). Higher LDL levels were associated with a greater reduction in SBP. Some studies have found that patients with higher preoperative LDL or total cholesterol levels tend to have higher baseline blood pressure, so more significant blood pressure reductions may be observed after RDN (24). A possible mechanism is that hyperlipidemia enhances sympathetic nervous system activity, which in turn affects blood pressure regulation, making the antihypertensive effect of RDN more pronounced.

The core mechanism of RDN is to reduce renal sympathetic nervous activity (25), thereby lowering blood pressure. In the state of hypertension, the overactivity of the sympathetic nervous system not only increases small vessel resistance but can also regulate microcirculatory pressure and alter microcirculatory resistance through selective responses in large arteries (26, 27). The NO released by the parasympathetic nervous system, as an important factor in regulating vascular dilation, effectively counterbalances the constrictive effects of the sympathetic nervous system (28, 29). Our study found a positive correlation between the reduction in IMR and the extent of blood pressure reduction, suggesting that improvements in microcirculatory resistance may play an important role in the antihypertensive mechanism of RDN.

In the vast field of exploring coronary artery physiological assessment, Coronary Flow Reserve (CFR) and Fractional Flow Reserve (FFR) focus on the overall functional assessment of the coronary arteries (30), while the IMR, as a new indicator, is mainly used to evaluate coronary microcirculatory disorders. The traditional method of measuring IMR with a pressure wire is clinically challenging due to its time, cost, and technical difficulty, as well as its association with adverse reactions to vasodilators. Therefore, an increasing number of studies are using the Coronary Angiography-based Index of Microcirculatory Resistance (caIMR) as an alternative to pressure wire-measured IMR, and the two show excellent diagnostic concordance (10, 31). Currently, FFR and IMR are mainly applied in the renal artery field for the interventional treatment of renal artery stenosis, with a recent sub-study of the FAIR-pilot study presented at the European Cardiology Conference that combines the use of FFR and IMR to predict the prognosis of interventional treatment for renal artery stenosis (32).

Currently, many RDN procedures rely on real-time monitoring of impedance to guide the ablation process, i.e., ablation is stopped when the impedance drop at each ablation site reaches a predetermined threshold (such as 10%–15%), which can control the extent and range of ablation (33). However, this method faces significant limitations, mainly the natural differences in impedance between renal artery branches and the inability of the absolute drop value to be directly converted into a relative percentage. In addition, impedance changes may also be affected by various non-ablation factors, such as vascular spasm and hemodynamic fluctuations, which increase the difficulty of interpreting the results. This difference makes it difficult to standardize the impedance-based ablation endpoints across different patients, thus affecting the precision and reproducibility of procedure outcomes. In contrast, IMR, as an indicator that directly reflects the resistance of renal artery microcirculation, has the advantage of being able to assess the activity state of the renal sympathetic nerves more directly and accurately. IMR is not interfered with by factors such as the underlying disease, age, and gender, providing a relatively objective and stable assessment standard. Moreover, the detection process of renal artery IMR (raIMR) is simple, non-invasive, and easy to be promoted and applied in clinical practice. Through preoperative and postoperative IMR monitoring, doctors can evaluate the immediate effects and long-term trends of ablation more accurately, thus formulating more personalized and precise RDN treatment strategies. According to our model validation, if the calMR results after renal artery ablation do not meet expectations, it indicates insufficient renal denervation and may require additional ablation points to achieve more comprehensive renal denervation treatment.

Despite the demonstrated superiority of the Ridge regression model in predicting blood pressure changes after RDN, this study has certain limitations. First, the relatively small sample size and single-center design may introduce selection bias and limit the generalizability of our findings. Therefore, validation in larger, multicenter cohorts is warranted, and external validation is necessary to confirm these preliminary results and establish their broader applicability. Second, although we considered multiple potential predictors, we were unable to include all other possible factors that would influence blood pressure. Future research could further optimize the model by incorporating more clinical features and external validation, thereby improving its predictive performance. Additionally, with the development of machine learning techniques, other advanced modeling methods might lead to new breakthroughs in this field.

## Conclusion

This study constructed and compared two prediction models (OLS regression model and Ridge regression model) to predict blood pressure changes 6 months after RDN based on clinical indicators in patients with resistant hypertension. Both models indicated that IMR was a factor associated with postoperative blood pressure reduction. Patients with a larger decrease in IMR after the procedure tended to show a greater reduction in blood pressure. The Ridge regression model outperformed the OLS model in terms of predictive performance and stability. These findings lay the foundation for future application and also suggest that further optimization of model parameters and integration of more clinically relevant features could enhance predictive performance, thereby better guiding the design and implementation of personalized treatment plans.

## Data Availability

The raw data supporting the conclusions of this article will be made available by the authors, without undue reservation.
