# Supplementary material for: A clinical prediction model for blood pressure changes after renal denervation in patients with resistant hypertension
Source: Front Cardiovasc Med. 2025 Jul 21;12:1637388. doi: 10.3389/fcvm.2025.1637388 (PMC12319003; doi:10.3389/fcvm.2025.1637388)
Supplement: Supplementary file 1 [file Table1.pdf]

Supp table 1. Change in SBP and DBP post six months according to selected categorical variables

Variables	SBP change		DBP change	
	mean ± SD	P value	mean ± SD	P value
Gender				
Male	-13.6±17.03	0.869	-5.6±8.73	0.636
Female	-12.8±14.93		-4.5±6.50	
Coronary heart disease				
Yes	-9.5±16.82	0.073	-4.0±8.08	0.214
No	-16.7±15.69		-6.5±8.35	
Myocardial infarction				
Yes	-9.5±18.07	0.144	-4.2±8.84	0.380
No	-15.6±15.34		-6.0±7.96	
Cerebral infarction				
Yes	2.3±6.12	0.0001	1.3±4.84	0.213
No	-15.0±16.39		-5.8±8.44	
Smoking				
Yes	-8.61±16.14	0.149	-3.8±7.77	0.362
No	-15.2±16.42		-5.9±8.44	
