# Supplementary material for: A clinical prediction model for blood pressure changes after renal denervation in patients with resistant hypertension
Source: Front Cardiovasc Med. 2025 Jul 21;12:1637388. doi: 10.3389/fcvm.2025.1637388 (PMC12319003; doi:10.3389/fcvm.2025.1637388)
Supplement: Supplementary file 2 [file Table2.pdf]

Supp table 2. Definitions of selected continuous variables and their linear relationship with changes in SBP and DBP at 6 months

Variables	Definitions	SBP change		DBP change	
		R squared	P values	R squared	P values
Age (years)	/	0.011	0.385	0.115	0.004
raIMR (Pre-LRA)	Preoperative left renal artery microcirculatory resistance index	0.061	0.041	0.076	0.021
raIMR (Post-LRA)	Postoperative left renal artery microcirculatory resistance index	0.151	0.001	0.060	0.043
raIMR (LRA)	Left renal artery microcirculatory resistance index (postoperative - preoperative)	0.313	<0.0001	0.203	0.0001
raIMR (Pre-RRA)	Preoperative right renal artery microcirculatory resistance index	0.066	0.033	0.071	0.026
raIMR (Post-RRA)	Postoperative right renal artery microcirculatory resistance index	0.091	0.012	0.045	0.080
raIMR(RRA)	Right renal artery microcirculatory resistance index (postoperative - preoperative)	0.202	0.0001	0.158	0.0007

Variables	Definitions	SBP change		DBP change	
		R squared	P values	R squared	P values
IMR	caIMR(LRA)+ caIMR(RRA)	0.456	<0.0001	0.323	<0.0001
First renal artery (Pre-LRA) (mm)	Preoperative diameter of the first left renal artery lumen	0.037	0.133	0.007	0.529
First renal artery (Post-LRA) (mm)	Postoperative diameter of the first left renal artery lumen	0.045	0.095	0.008	0.490
First renal artery (LRA) (mm)	Change in lumen diameter of the left first renal artery	0.013	0.382	0.002	0.736
Second renal artery (Pre-LRA) (mm)	Preoperative diameter of the second left renal artery lumen	0.047	0.086	0.007	0.517
Second renal artery (Post-LRA) (mm)	Postoperative diameter of the second left renal artery lumen	0.048	0.084	0.008	0.493
Second renal artery (LRA) (mm)	Change in lumen diameter of the left second renal artery	0.001	0.785	0	0.989

Variables	Definitions	SBP change		DBP change	
		R squared	P values	R squared	P values
First renal artery (Pre-RRA) (mm)	Preoperative diameter of the first right renal artery lumen	0.028	0.190	0	0.919
First renal artery (Post-RRA) (mm)	Postoperative diameter of the first right renal artery lumen	0.029	0.179	0.001	0.774
First renal artery (RRA) (mm)	Change in lumen diameter of the right first renal artery	0.011	0.406	0.001	0.778
Second renal artery (Pre-RRA) (mm)	Preoperative diameter of the second right renal artery lumen	0.036	0.137	0.002	0.738
Second renal artery (Post-RRA) (mm)	Postoperative diameter of the second right renal artery lumen	0.036	0.134	0.004	0.631
Second renal artery (RRA) (mm)	Change in lumen diameter of the right second renal artery	0.011	0.415	0.003	0.697
Pre-SBP (mmHg)	Preoperative systolic blood pressure	0.203	0.0001	0.031	0.145

Variables	Definitions	SBP change		DBP change	
		R squared	P values	R squared	P values
Post-SBP (mmHg)	Postoperative systolic blood pressure	0.011	0.387	0.018	0.274
Pre-DBP (mmHg)	Preoperative diastolic blood pressure	0.068	0.030	0.391	<0.0001
Post -DBP (mmHg)	Postoperative diastolic blood pressure	0.003	0.633	0.001	0.844
White blood cell (10^9/L)	Preoperative value	0.011	0.406	0	0.985
Platelets (10^9/L)	Preoperative value	0.001	0.768	0.004	0.639
Haemoglobin (g/L)	Preoperative value	0.016	0.317	0.010	0.431
Alanine transaminase (U/L)	Preoperative value	0.034	0.132	0.006	0.517
Aspartate transferase (U/L)	Preoperative value	0.071	0.046	0.015	0.336
Creatinine (μmol/L)	Preoperative value	0.061	0.042	0.058	0.047
Uric acid (μmol/L)	Preoperative value	0.002	0.740	0.004	0.629
Blood urea nitrogen (mmol/L)	Preoperative value	0.001	0.779	0.006	0.523
Glucose (mmol/L)	Preoperative value	0.002	0.756	0.016	0.351
Glycated haemoglobin (%)	Preoperative value	0.015	0.429	0.002	0.771

Variables	Definitions	SBP change		DBP change	
		R squared	P values	R squared	P values
Total cholesterol (mmol/L)	Preoperative value	0.087	0.014	0.087	0.014
Triglyceride (mmol/L)	Preoperative value	0.017	0.348	0.040	0.146
Low-density Lipoprotein (mmol/L)	Preoperative value	0.113	0.005	0.072	0.026
High-density Lipoprotein (mmol/L)	Preoperative value	0.050	0.105	0.049	0.109
Left ventricular ejection fraction (%)	Preoperative value	0.002	0.715	0.004	0.635
